# Early Postoperative Physical Frailty Reflects Functional Vulnerability and Predicts Prolonged Hospitalization After Major Cardiovascular Surgery

**DOI:** 10.3390/life16030395

**Published:** 2026-02-28

**Authors:** Seoyon Yang, Younji Kim, Suk-Won Song, Ha Lee, Myeong Su Kim, You Gyoung Yi

**Affiliations:** 1 Department of Rehabilitation Medicine, Ewha Womans University Seoul Hospital, College of Medicine, Ewha Womans University, Seoul 07804, Republic of Korea; seoyonyang@gmail.com (S.Y.); yunji0114@naver.com (Y.K.); 2 Department of Thoracic and Cardiovascular Surgery, Ewha Womans University Aorta and Vascular Hospital, Ewha Womans University Medical Center, Seoul 07985, Republic of Korea; stevensong@ewha.ac.kr (S.-W.S.); manofwill@naver.com (H.L.); mmotion11@ewha.ac.kr (M.S.K.)

**Keywords:** frailty, physical function, cardiac surgery, aortic surgery, length of stay, rehabilitation

## Abstract

**Background:** Although frailty has emerged as an important determinant of outcomes following cardiovascular surgery, the clinical significance of early postoperative physical frailty assessed during the acute recovery phase has not been investigated. **Methods:** We conducted a single-center retrospective observational study including patients who underwent cardiac or aortic surgery and completed a standardized physical function assessment within 10 days postoperatively. Physical frailty was defined using four objective indicators: Medical Research Council (MRC) sum score, gait speed, Timed Up and Go test, and five-times sit-to-stand test. Frailty was defined as the presence of ≥3 abnormal physical frailty indicators. Clinical outcomes included hospital length of stay (LOS) and postoperative medical complications. Negative binomial regression was used to evaluate factors associated with hospital LOS. **Results:** Among 441 patients included in the analysis, 308 (69.8%) were classified as frail. Frail patients were older and demonstrated significantly impaired physical performance across all frailty indicators (all *p* < 0.001). Frailty was associated with longer ICU stay and hospital LOS (both *p* < 0.001). In multivariable negative binomial regression, postoperative frailty was independently associated with prolonged hospital LOS (incidence rate ratio [IRR] 1.38, 95% CI 1.26–1.51; *p* < 0.001), after adjustment for age and timing of frailty assessment. Additional adjustment for surgical approach and surgical target did not improve model fit. Postoperative frailty was not significantly associated with the overall incidence of medical complications. **Conclusions**: Early postoperative physical frailty, assessed during the acute recovery phase, is independently associated with prolonged hospitalization after cardiac and aortic surgery. These findings suggest that early functional vulnerability captures clinically meaningful risk beyond surgical characteristics and may serve as a valuable target for postoperative risk stratification and rehabilitation planning.

## 1. Introduction

Frailty, a syndrome of diminished physiologic reserve and increased vulnerability has emerged as an important determinant of outcomes in older adults undergoing major cardiovascular surgery [1]. Patients identified as frail prior to cardiac or aortic operations consistently experience higher rates of postoperative mortality, complications, and functional decline [1]. It has been reported that frailty indicates a unique vulnerability to surgical stress not accounted for by traditional risk models alone. Assessing frailty before surgery has therefore become a focus for improving risk stratification in cardiac care [2].

Simple physical performance tests—such as the 5-m gait speed, handgrip strength, Timed Up-and-Go, or chair-stand tests—serve as validated frailty measures and independently predict surgical outcomes. Notably, gait speed has proven to be a powerful prognostic indicator: each 0.1 m/s decrease in walking speed is associated with an ~11% relative increase in operative mortality risk after cardiac surgery [2]. Incorporating such frailty metrics into preoperative risk assessments significantly improves the identification of high-risk patients. Indeed, large cohort studies show that frail patients tend to have longer intensive care and hospital stays and are more often discharged to rehabilitation or nursing facilities [3]. These findings emphasize the clinical importance of frailty as a risk marker and have led to efforts to routinely assess frailty in candidates for cardiac surgery.

While prior studies have consistently highlighted the prognostic importance of preoperative frailty in cardiac or transcatheter procedures [4,5,6], few have evaluated physical frailty during the acute postoperative phase. The concept of “acute postoperative frailty” in older patients was recognized over a decade ago [7], yet concrete evidence of its prognostic implications is limited. Early after major surgery, patients often exhibit significant declines in strength and mobility due to the stress of surgery, bed rest, and perioperative complications [8]. Despite the growing recognition of frailty as a predictor of poor outcomes, it remains uncertain whether formal assessment of physical frailty in the early postoperative period—within the first few days after cardiac or aortic surgery—offers independent prognostic value beyond preoperative comorbidities and intraoperative factors.

In the present study, we aimed to investigate the clinical significance of early postoperative physical frailty during the acute recovery phase following major cardiovascular surgery. We conducted objective frailty assessments within the first 10 postoperative days using standardized physical performance measures. Our primary objective was to evaluate whether early postoperative frailty is independently associated with hospital length of stay (LOS) and adverse outcomes. Furthermore, we aimed to identify other independent predictors of hospital LOS through multivariable regression analysis, to investigate the relative contribution of frailty adjusting for conventional demographic and clinical factors.

## 2. Materials and Methods

### 2.1. Study Design and Population

This was a retrospective observational study conducted at Ewha Woman’s University Seoul Hospital. We reviewed electronic medical records of adult patients who underwent major cardiovascular surgery, including cardiac and thoracic aortic procedures, between November 2024 and December 2025. Eligible patients were those who completed a standardized physical frailty assessment within the first 10 postoperative days, corresponding to the early recovery phase.

Patients were excluded if they experienced in-hospital mortality prior to assessment, had pre-existing neurological conditions that would interfere with physical function testing, experienced major postoperative neurological complications, or completed frailty assessments beyond postoperative day (POD) 10. The rationale for excluding patients with late assessments (POD > 10) was based on the need to evaluate frailty as a potential early prognostic marker, distinct from complications or secondary declines in function. Assessments performed beyond this acute window may reflect interim events rather than intrinsic vulnerability, thereby confounding interpretation. Moreover, standardized rehabilitation protocols and clinical decision-making typically occur within the first 10 days post-surgery, increasing the clinical relevance of early frailty identification.

### 2.2. Ethics Approval

The study protocol was reviewed and approved by the Institutional Review Board of Ewha Woman’s University Seoul Hospital (IRB No. SEUMC 2025-12-059-001). Given the retrospective nature of this study, informed consent was waived. Data collection was conducted in accordance with the principles of the Declaration of Helsinki and institutional ethical standards.

### 2.3. Frailty Assessment

Physical frailty was assessed using four objective indicators: gait speed, Timed Up and Go test, five-times sit-to-stand (5STS) test, and Medical Research Council (MRC) sum score. Each component was dichotomized based on established clinical cut-offs (gait speed < 0.6 m/s [9], TUG > 12 s [10], 5STS > 15 s [11,12], and MRC sum score < 48 [13,14]). A frailty score ranging from 0 to 4 was calculated, with frailty defined as the presence of ≥3 abnormal indicators.

### 2.4. Data Collection

Clinical and demographic variables were extracted from electronic medical records, including age, sex, body mass index (BMI), comorbidities (e.g., hypertension, diabetes mellitus, chronic kidney disease), surgical target and approach, timing of frailty assessment, and outcomes. The primary outcome was total hospital length of stay (LOS). Secondary outcomes included ICU days and the incidence of postoperative medical complications. Data extraction and coding were independently verified by two clinical investigators.

### 2.5. Statistical Analyses

Descriptive statistics were used to summarize baseline characteristics by frailty status. Continuous variables were analyzed using *t*-tests or Wilcoxon rank-sum tests, and categorical variables with chi-square or Fisher’s exact tests, as appropriate. Multivariable negative binomial regression was used to assess the association between postoperative frailty and hospital LOS, adjusting for potential confounders. The strength of associations was expressed as incidence rate ratios (IRRs) with 95% confidence intervals (CIs). Model performance was evaluated using Akaike Information Criterion (AIC). Logistic regression was used to explore associations with postoperative complications. All statistical analyses were performed using R version 4.5.2 and a two-sided *p*-value < 0.05 was considered statistically significant.

## 3. Results

### 3.1. Study Participants and Characteristics

**Figure 1:**
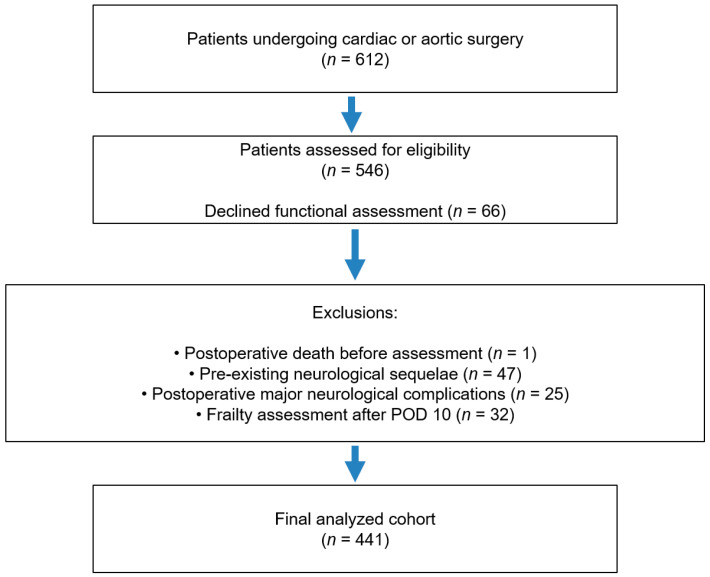
Flow diagram of patient selection for postoperative frailty assessment after cardiac or aortic surgery.

During the study period, 612 patients underwent cardiac or aortic surgery at our institution (Figure 1). Among them, 66 patients declined postoperative rehabilitation or functional assessment and were excluded. Of the remaining 546 patients, 1 patient died before frailty assessment, 47 had pre-existing neurological sequelae, 25 experienced major postoperative neurological complications (including spinal cord injury, intracranial hemorrhage, or cerebral infarction), and 32 underwent frailty assessment beyond postoperative day 10. Consequently, 441 patients were included in the final analysis cohort.

Patients were classified into frail and non-frail groups based on their postoperative physical frailty status. Specifically, frailty was defined as the presence of three or more abnormal physical frailty indicators, as described in Methods Section 2.3, while patients with zero to two abnormal indicators were classified as non-frail.

Among the total cohort of 441 patients who underwent postoperative frailty assessment (Figure 1), 133 (30%) were classified as non-frail and 308 (70%) as frail (Table 1). Frail patients were significantly older than non-frail group, with a mean age of 69 ± 14 years compared to 61 ± 14 years in the non-frail group (*p* < 0.001). A significantly higher proportion of frail patients were female (45% vs. 17%, *p* < 0.001). There were no significant differences between frail and non-frail patients in baseline comorbidities, including body mass index (BMI), hypertension, diabetes mellitus, chronic kidney disease, chronic obstructive pulmonary disease, or congestive heart failure (all *p* > 0.05). The type of surgical approach (open vs. endovascular) was also comparable between groups (*p* = 0.7).

The timing of frailty assessment was standardized to perform within the early postoperative period, with a median assessment day of postoperative day (POD) 7 (interquartile range [IQR], 5.0–8.0), and no significant difference in timing between frail and non-frail groups (*p* = 0.6). This consistency strengthens the internal validity of the frailty classification and minimizes potential bias due to variability in recovery timeframes.

Significant differences were observed between frail and non-frail patients across all four physical performance indicators (Table 2). However, it is noteworthy that these differences were not limited to a single component. Rather, frail patients exhibited consistent and marked impairments across every individual measure, suggesting a more global decline in physical function.

The median MRC sum score, reflecting overall muscle strength, was substantially lower in frail patients (36.00 [IQR: 36.00–42.00]) than in non-frail counterparts (48.00 [IQR: 42.00–48.00], *p* < 0.001). Gait speed, a robust marker of functional mobility, was significantly slower among frail patients (0.28 m/s [IQR: 0.00–0.47]) compared to non-frail patients (0.73 m/s [IQR: 0.61–0.90], *p* < 0.001). Similarly, time-based mobility tests revealed pronounced impairments: the Timed Up and Go test was prolonged in the frail group (24.50 s [IQR: 19.50–31.80]) versus the non-frail group (12.00 s [IQR: 9.40–15.37], *p* < 0.001), and the 5-times sit-to-stand (5STS) test showed comparable trends (20.35 s [IQR: 15.80–25.40] vs. 11.00 s [IQR: 8.80–13.00], *p* < 0.001). Patients classified as frail exhibited impairments across all physical performance domains, reflecting a generalized postoperative decline in neuromuscular and functional capacity.

### 3.2. Comparison of Clinical Outcomes and Postoperative Complications Between Non-Frail and Frail Group

Postoperative clinical outcomes differed markedly between frail and non-frail patients (Table 3), particularly in intensive care unit (ICU) and hospital LOS. Frail group experienced significantly longer ICU stays, with a median duration of 3.0 days (interquartile range [IQR]: 1.5–5.0), compared to 2.0 days (IQR: 1.0–3.0) in the non-frail group (*p* < 0.001). A similar trend was observed for overall hospital length of stay, where the frail cohort had a median hospitalization of 24.0 days (IQR: 17.0–34.0), which was significantly longer than the 17.0 days (IQR: 12.0–24.0) observed in the non-frail group (*p* < 0.001).

In contrast, the overall incidence of postoperative medical complications was relatively low and did not differ meaningfully between groups, with complication rates of 10% in both frail and non-frail patients (*p* > 0.9). Specific complications such as acute kidney injury (AKI), clinically significant arrhythmias, and respiratory failure or the need for reintubation were rare across the cohort.

### 3.3. Association Between Frailty and Hospital Length of Stay

To evaluate the independent association between early postoperative frailty and hospital LOS, a multivariable negative binomial regression analysis was performed as shown in Table 4. The analysis revealed that patients classified as frail had significantly longer hospitalizations, with an incidence rate ratio (IRR) of 1.38 (95% confidence interval [CI]: 1.26–1.51; *p* < 0.001), even after adjusting for potential confounding factors. In addition to frailty status, older age (IRR 1.01, 95% CI: not specified; *p* < 0.001) and a later postoperative day of frailty assessment (IRR 1.07, 95% CI: not specified; *p* < 0.001) were independently associated with increased LOS.

Model fit was evaluated using Akaike’s Information Criterion (AIC) to compare the explanatory power of the minimally adjusted model (which included age and frailty assessment timing) versus a fully adjusted model (which additionally incorporated surgical approach and target anatomy). The minimally adjusted model yielded a lower AIC (3214.4) compared to the fully adjusted model (3216.6), indicating no improvement in model fit with the inclusion of surgical variables.

## 4. Discussion

### 4.1. Study Participants and Characteristics

In this cohort of cardiac and aortic surgery patients, we found a notably high prevalence of postoperative frailty (70%), reflecting the significant impact of major surgery on early functional status. This frailty rate is higher than typical preoperative frailty estimates (often 20–40% in cardiac surgery patients) [15], likely because our assessment captured acute post-surgical deconditioning. Frail patients were on average 8 years older than non-frail, consistent with the strong association of advanced age and frailty observed in general and surgical populations [16]. We also observed a striking sex difference: nearly half of frail patients were female, versus only 17% in the non-frail group. This aligns with epidemiologic data that frailty is more prevalent in older women than men [16], potentially due to differences in longevity, muscle mass, and other factors. Importantly, baseline comorbidities (e.g., hypertension, diabetes, COPD) did not significantly differ between frail and non-frail groups, reinforcing that frailty is a distinct domain of risk not fully captured by traditional comorbidity counts. Even with comparable comorbidity burdens, older patients (especially women) may have reduced physiological reserve and strength, predisposing them to postoperative frailty [15,16]. These findings highlights the value of formal frailty assessments, as standard risk factors alone may overlook patients with diminished functional reserve.

The timing of frailty evaluation was standardized (median postoperative day 7 for both groups), which strengthens internal validity. By assessing all patients at a similar early postoperative interval, we ensured that the observed differences in frailty status were not due to variations in recovery time. This approach captures the early postoperative frailty state—a combination of preoperative vulnerability and acute surgery-related decline. Notably, even patients who were functionally independent before surgery can develop significant frailty symptoms in the first week after a major cardiac operation due to bedrest, inflammation, and stress responses. The fact that such a large proportion met frailty criteria postoperatively highlights how universal early functional impairment is after complex cardiac procedures, even in patients with similar pre-surgical comorbid profiles. Our frailty classification within 10 days of surgery appears to delineate a vulnerable subset of patients who are older and predominantly female, consistent with known risk factors for frailty [16]. The threshold of ≥3 abnormal indicators was selected to reflect a robust frailty phenotype, consistent with prior studies using multiple physical performance measures in surgical populations [17]. While this cutoff may contribute to a higher observed prevalence, it was chosen to capture patients with clinically meaningful functional vulnerability in the early postoperative period. This emphasizes that chronological age and sex are important but not exclusive determinants—objective functional testing is needed to identify frailty in postoperative patients who might otherwise be overlooked if one relies only on age or medical history.

Objective tests of physical function revealed stark differences between frail and non-frail patients in the first 10 days after surgery, confirming the clinical significance of our frailty classification. The frail group had a median Medical Research Council (MRC) sum score of 36, versus 48 in non-frail patients—a dramatic gap in muscle strength. An MRC sum score below 48/60 is widely considered indicative of clinically significant weakness (consistent with intensive care unit-acquired weakness), and scores below 36 denote severe weakness [18]. Thus, the frail cohort’s median score of 36 falls at the threshold of severe global weakness, whereas the non-frail median of 48 represents the lower limit of normal strength. This suggests that many “non-frail” patients still had some weakness, but the frail patients were profoundly weaker, likely reflecting postoperative critical illness myopathy and deconditioning. Prior studies have shown that patients with MRC scores < 48 are at high risk for prolonged ventilation, extended ICU stays, and increased mortality [18], which aligns with our finding in subsequent sections that the frail group had longer critical care needs. The substantial early strength deficit in frail patients highlights the impact of acute sarcopenia and bed rest in the postoperative period.

Mobility assessments similarly demonstrated substantial impairments among frail patients. Frail individuals had a median gait speed of only 0.28 m/s, compared to 0.73 m/s in the non-frail group (who were close to a normal elderly gait speed). A gait speed of ~0.8 m/s is often used as a frailty cutoff in cardiac surgery patients [15], and slower gait is a well-established predictor of worse outcomes. Indeed, gait speeds below 0.83 m/s have been associated with higher operative mortality risk in cardiac surgery [15]. The frail group’s gait speed (0.28 m/s) is extremely slow—many were likely unable to walk unassisted (the IQR included 0 m/s, indicating some patients could not perform the test). Such profound slow gait or inability to ambulate in the first postoperative week signifies severe functional dependence.

Time-based functional tests further illustrate the gap in functional performance. The TUG test median was 24.5 s in frail patients versus 12.0 s in non-frail. In geriatric mobility assessment, a TUG time over ~20 s indicates significant mobility impairment and risk of falls or need for assistance [19]. Our frail cohort far exceeded this threshold (many took well over 20 s, or could not complete the TUG at all), whereas non-frail patients were around 12 s, which is within normal limits for older adults. This two-fold difference in TUG underscores that frail patients had marked deficits in balance and walking ability early after surgery. Similarly, the 5STS test median was ~20.3 s in frail patients versus 11.0 s in non-frail. For older adults, 5STS times > 15 s are indicative of lower extremity weakness and predictive of future disability. Thus, the frail group’s performance was poor, reflecting reduced leg strength and endurance, whereas the non-frail group’s 11-s time approaches normal age-matched performance. Taken together, these objective measures confirm that frail patients experienced a substantial loss of physical function across multiple domains in the immediate postoperative period.

It is noteworthy that these functional impairments were measured at a uniform early time point (around one week post-surgery). This suggests that early postoperative frailty captured by tests like gait speed, TUG, and 5STS is not simply a transient phenomenon but rather a meaningful indicator of patients’ physiologic reserve. Many frail patients in our study likely met criteria for intensive care unit-acquired weakness and post-operative functional decline. The literature supports that even a week of hospitalization with limited mobility can lead to significant muscle atrophy and weakness, especially in older adults [20]. Our findings mirror this: despite similar comorbid profiles, frail patients exhibited the hallmarks of acute functional decline. This has important clinical implications—such severe weakness and slowness early on could predispose patients to downstream complications (e.g., falls, delirium) and delay their recovery trajectory. Identifying these deficits promptly enables targeted interventions (physical therapy, mobilization protocols, nutrition supplementation) to prevent further decline. Indeed, gait speed and other physical performance tests are increasingly recognized as valuable components of frailty assessment in cardiac surgery. The pronounced differences we observed validate the use of these simple bedside tests to stratify risk: a patient unable to walk or taking >20–30 s for TUG on postoperative day 7 clearly has a different recovery outlook than one who is walking 0.8 m/s and completing TUG in 12 s.

### 4.2. Comparison of Clinical Outcomes and Postoperative Complications Between Non-Frail and Frail Group

Frail patients had significantly worse clinical outcomes during the postoperative course, even though major complication rates were statistically similar between groups. One of the clearest disparities was in length of stay. Patients classified as frail spent a median of 3 days in the intensive care unit (ICU) compared to 2 days for non-frail patients, and their total hospital stay was a median of 24 days versus 17 days in the non-frail group. These differences (roughly 50% longer ICU stay and 40% longer total hospitalization for frail patients) are clinically substantial. They remained significant even after adjusting for age and other factors. Our findings are in line with a growing body of literature that links frailty to prolonged recovery and resource utilization after surgery. Multiple studies in cardiac surgery populations have documented that frail patient require longer ICU care and extended hospital stays [15]. For example, frailty (defined by various tools such as the Clinical Frailty Scale or Frailty Index) has been correlated with increased ICU days after CABG or valve surgery in several cohorts [1,3,4,6]. Similarly, frailty has been associated with a higher likelihood of protracted hospital stays, including difficulty achieving discharge before 2 weeks postoperatively. This is consistent with the concept that reduced physiologic reserve in frailty leads to slower convalescence—tasks like weaning from ventilator support, mobilizing out of bed, and regaining independence take longer, translating into extra days in critical care and on the ward.

Interestingly, the overall incidence of postoperative medical complications (e.g., acute kidney injury, significant arrhythmias, or respiratory failure requiring reintubation) was low in our cohort (around 10%) and did not differ significantly between frail and non-frail groups. This contrasts with many previous studies that have found frailty to be associated with higher complication rates. Meta-analyses encompassing tens of thousands of cardiac surgery patients report that frail individuals are at elevated risk for major adverse events such as stroke, renal failure, prolonged ventilation, and infectious complications. For instance, Lee et al. found frailty associated with increased odds of perioperative stroke and sternal wound complications in cardiac surgery patients [6]. Another recent meta-analysis noted that frail patients had higher incidence of delirium, prolonged mechanical ventilation > 48 h, acute kidney injury, and reoperation for bleeding [21]. Our study did not observe such differences, which likely reflects a combination of our cohort’s characteristics and study design. We excluded patients with major neurological complications (stroke, spinal cord injury) from the analysis, as well as the single early postoperative death, which removed some of the most severe adverse events from comparison. Additionally, our sample size (*n* = 441) and the overall low event rates mean we may have been underpowered to detect modest differences in complication incidence. It is worth noting that other smaller single-center studies have similarly reported no significant difference in certain complications between frail and non-frail patients. Ad et al. [22] found that frailty (defined by Cardiovascular Health Study criteria) did not predict stroke, atrial fibrillation, or renal failure in a cohort of 167 cardiac surgery patients, attributing this to limited sample size. In our cohort, the lack of divergence in complications might also indicate that with modern perioperative care, frail patients can be medically managed to avoid some complications, but they still experience an extended recovery time. In other words, frailty in our population manifested more in slower recovery and functional dependency rather than discrete medical complications.

Despite similar complication rates, frail patients experienced significantly longer ICU and hospital stays, underscoring the substantial healthcare impact of frailty. Prolonged hospitalization increases costs and exposes patients to risks such as hospital-acquired infections and deconditioning. Frail patients also more frequently required post-acute care after discharge, whereas non-frail patients were more likely to return home. Although discharge disposition was not explicitly reported in our study, the prior literature consistently shows that frail cardiac surgery patients have higher rates of discharge to skilled nursing or rehabilitation facilities. A meta-analysis by Wong et al. demonstrated that frail individuals are far more likely to require institutional care and experience hospital readmissions after cardiac surgery [23,24], reflecting greater dependency [23] and ongoing care needs. Overall, these findings suggest that frailty prolongs recovery and necessitates extended support beyond the acute hospital setting, even in the absence of increased surgical complications.

### 4.3. Frailty as an Independent Predictor of Prolonged Hospital Stay

After adjustment for covariates, frailty was independently associated with a significantly prolonged hospital stay. Older age showed a modest effect (IRR ~1.01 per year, *p* < 0.001). The postoperative day of frailty assessment was also an independent predictor (IRR ~1.07 per day delay, *p* < 0.001), indicating that later assessment—likely reflecting slower early recovery—was associated with longer length of stay. Notably, adding operative variables (e.g., surgical approach or procedure type) did not improve model performance, suggesting that the effect of frailty on length of stay was independent of surgical complexity. Consistent with prior large-scale analyses, frailty remained a robust predictor of prolonged hospitalization regardless of procedure type, and our findings extend this association to frailty assessed in the early postoperative period [25].

These findings highlight that postoperative frailty captures a risk dimension not fully accounted for by age or surgical factors. A 75-year-old frail patient and a 75-year-old fit patient undergoing the same cardiac procedure have markedly different recovery trajectories, even if their surgeries are uneventful. Frailty likely integrates the effects of subtle vulnerabilities—sarcopenia, poor endurance, cognitive impairment, etc.—that influence how quickly a patient can be liberated from intensive support and achieve milestones like ambulation or self-care. Our multivariable analysis supports frailty as an independent marker for slower recovery. Ahuja et al. reported that increasing Clinical Frailty Scale scores were associated not only with higher mortality but also with significantly longer ICU and hospital stays, independent of other risk factors [3]. Likewise, we found an almost 40% extension in LOS attributable to frailty, even when holding age constant. It is important to highlight that age itself, while statistically significant, had a relatively small effect size (each additional year of age ~1% longer stay), whereas frailty status (which often correlates with age but not perfectly) had a much larger impact. This reinforces the idea that chronological age and biological frailty are related but distinct entities. Many octogenarians who are robust can recover quickly, whereas some patients in their 60 s who are frail may recover slowly—our model captures this by giving frailty its own weight.

Beyond statistical associations, the clinical implication is that early identification of frailty can help predict which patients will need prolonged inpatient care. This can inform bed management, discharge planning, and family counseling soon after surgery. For instance, a patient flagged as frail on postoperative day 5–7 is very likely to still be hospitalized a week or two later, whereas a non-frail patient might be ready for discharge. Recognizing this in advance allows the care team to arrange rehabilitation facility placement or additional home support as needed, potentially smoothing transitions of care. Moreover, understanding frailty’s independent role encourages clinicians to target modifiable factors within the hospital stay. For example, aggressive preventive measures like early physical therapy, nutritional supplementation, and delirium precautions might shorten the stay or at least improve outcomes for a frail patient. While our study did not evaluate the efficacy of specific interventions to reduce hospital LOS, it clearly identifies postoperative frailty as a robust marker of patients at risk for prolonged hospitalization without additional support. This predictive utility supports growing recommendations to incorporate routine frailty assessment into perioperative care planning and to proactively tailor inpatient management for vulnerable patients.

It is also worth noting that the day of frailty assessment being linked to LOS raises an interesting point: if a patient is progressing well, they are likely evaluated and mobilized earlier; if not, their very delay in functional assessment is a bad prognostic sign. This might be seen as a form of immobility time—every extra day that a patient remains too ill or weak to attempt functional tests likely compounds muscle loss and demoralization, further prolonging recovery. This underscores the importance of early mobilization: getting patients assessed and moving as soon as feasible could potentially break this cycle. In summary, our adjusted analysis confirms that postoperative frailty is not merely a bystander but a key driver of prolonged hospitalization, independent of age and operative variables. It strengthens the argument that frailty assessment should be integrated into postoperative care and that interventions should be directed at this high-risk group to improve efficiency of recovery.

Another implication is the potential value of “prehabilitation” for high-risk patients. Knowing that 70% of our cohort became frail post-surgery, one might ask if we can reduce that incidence by improving patients’ resilience before surgery. Prehabilitation programs, typically involving supervised exercise training, nutritional optimization, and education in the weeks leading up to surgery, have shown benefit in other settings [26,27,28,29]. In cardiac surgery, emerging evidence suggests that prehabilitation for frail or nearly frail patients can improve postoperative outcomes and shorten recovery time. For instance, aerobic exercise programs before surgery have been associated with reduced postoperative ventilation needs and shorter ICU stays. These efforts reflect a paradigm shift: rather than reactively dealing with frailty after surgery, proactively “defrailing” patients could improve their postoperative trajectory. Our findings—particularly that frailty independently prolongs hospital stay—provide real-world justification for such trials. If an intervention can reduce the prevalence or severity of frailty, it could directly translate into shorter hospitalizations and less ICU strain.

For patients who are identified as frail in the early postoperative period, the emphasis should be on accelerated rehabilitation and safe discharge planning. This includes daily physical therapy focusing on strength and balance, early nutritional support to counter catabolism, and delirium prevention strategies to maintain cognitive function. The goal is to prevent a vicious cycle of frailty worsening due to hospitalization itself. In older surgical patients, it is well documented that prolonged bed rest leads to compounding functional loss, increased risk of delirium, and institutionalization. Our frail patients, who already start behind in functional terms, are especially vulnerable to this hospital-associated decline. Thus, aggressive rehabilitation is not merely for eventual benefit—it is an acute treatment to improve the current hospitalization outcomes. There is reason for optimism that such approaches work. A randomized trial protocol in elderly emergency surgery patients hypothesized that a postoperative physical exercise program would prevent functional/cognitive decline and improve quality of life. While results are pending, the hypothesis (supported by pilot data) is that individualized, progressive exercise initiated soon after surgery can speed up recovery of mobility and independence. In the cardiac surgery realm, early mobilization protocols in the ICU have been associated with improved functional milestones and might reduce ICU delirium duration. Our data would encourage applying these rehabilitation principles as early as possible: for example, even on postoperative day 1 or 2, initiating bedside exercises or cycle ergometry (if feasible) might help frail patients retain muscle mass.

### 4.4. Limitations

A major limitation of this study is the lack of preoperative frailty assessment, which precludes clear distinction between baseline physiological vulnerability and postoperative functional decline. The frailty phenotype observed during early recovery likely reflects a combination of pre-existing limitations and acute deconditioning due to surgery and immobility. While this constrains causal inference, it mirrors common clinical settings in which patients are referred for rehabilitation without standardized preoperative frailty data. Future studies incorporating serial assessments from the pre- to postoperative period are warranted to delineate frailty trajectories and their relationship with outcomes. Additionally, our frailty definition (≥3 of 4 abnormal indicators) may have contributed to a higher observed prevalence and potential risk of overclassification, especially in the immediate postoperative period when temporary performance impairments are common. Future research is warranted to validate optimal cutoff points for early postoperative frailty and to differentiate transient deconditioning from persistent functional decline. Third, the observed low rate of medical complications may underestimate the true burden of postoperative morbidity. Patients who died early or developed major neurological complications were excluded, introducing potential selection and survivor bias. Moreover, other important outcomes such as delirium, discharge destination, or functional status at discharge were not uniformly captured and were therefore not analyzed. Future studies incorporating broader outcome measures and systematic postoperative assessments are needed to fully characterize the consequences of early frailty. Fourth, this was a single-center study conducted at a high-volume tertiary referral hospital where open cardiovascular surgery predominates. As such, the findings may not be generalizable to institutions with different patient populations or procedural approaches, such as centers with greater use of minimally invasive or transcatheter techniques. The frailty burden, recovery patterns, and postoperative care strategies may differ in those settings. Multicenter validation across diverse surgical practices is needed to confirm the broader applicability of these results.

## 5. Conclusions

In conclusion, the present study emphasize that frailty is a critical determinant of postoperative recovery, and they call for a proactive approach to perioperative care. By identifying frail patients early (within a week of surgery), we have an opportunity to initiate targeted interventions—“all hands on deck” supportive care involving rehabilitation therapists, geriatricians, nutritionists, and nursing—to improve outcomes. These patients may benefit from longer and more intensive physical therapy during hospitalization, early mobilization in ICU, and tailored discharge plans including rehabilitation services. The ultimate aim is to shorten the time to functional recovery and safe discharge, thereby reducing the prolonged hospital stays that frail patients currently experience. As the population of older adults undergoing cardiac and aortic surgery grows, integrating frailty assessment and management into standard practice will be essential. Our findings support ongoing efforts in both prehabilitation and postoperative rehabilitation to improve resilience in this vulnerable group. By linking our frailty assessments to actionable rehabilitation plans, we can hopefully translate the knowledge of “who is frail” into improved trajectories of recovery and independence for those patients.

## Figures and Tables

**Table 1:** Baseline Characteristics of Patients According to Postoperative Frailty Status.

Characteristic	Non-Frail Group (*N* = 133)	Frail Group (*N* = 308)	Total (*N* = 441)	*p*-Value ^2^
Age ^1^	61 ± 14	69 ± 14	67 ± 14	<0.001
Male Sex	110 (83%)	168 (55%)	278 (63%)	<0.001
Body Mass Index ^1^	24.7 ± 3.3	24.7 ± 4.6	24.7 ± 4.2	0.9
Hypertension	84 (63%)	194 (63%)	278 (63%)	>0.9
Diabetes	19 (14%)	61 (20%)	80 (18%)	0.2
Chronic Kidney Disease	5 (3.8%)	10 (3.2%)	15 (3.4%)	0.8
Chronic Obstructive Pulmonary Disease	3 (2.3%)	6 (1.9%)	9 (2.0%)	>0.9
Congestive Heart Failure	2 (1.5%)	7 (2.3%)	9 (2.0%)	0.7
Surgical Approach (Open)	132 (99%)	302 (98%)	434 (98%)	0.7
Postoperative Day of Frailty Assessment ^1^	7.00 (6.00, 7.00)	7.00 (5.00, 8.00)	7.00 (5.00, 8.00)	0.6

^1^ Values are presented as mean ± standard deviation (SD) or median (Q1, Q3) as appropriate. ^2^ Statistical tests used: Welch’s two-sample *t*-test for normally distributed continuous variables; Wilcoxon rank-sum test for non-normally distributed continuous variables; Chi-squared test or Fisher’s exact test for categorical variables, as appropriate.

**Table 2:** Postoperative Physical Frailty Indicators Assessed Within 10 Days After Surgery.

Characteristic	Non-Frail Group (N = 133)	Frail Group (N = 308)	Total (N = 441)	*p*-Value ^1^
MRC sum score ^2^	48.00 (42.00, 48.00)	36.00 (36.00, 42.00)	36.00 (36.00, 47.50)	<0.001
Gait speed (m/s)	0.73 (0.61, 0.90)	0.28 (0.00, 0.47)	0.42 (0.11, 0.63)	<0.001
Timed Up and Go (s)	12.00 (9.40, 15.37)	24.50 (19.50, 31.80)	19.55 (13.10, 28.40)	<0.001
Five times sit-to-stand (s)	11.00 (8.80, 13.00)	20.35 (15.80, 25.40)	16.00 (11.70, 22.70)	<0.001

^1^ Values are presented as median (Q1, Q3). ^2^ Wilcoxon rank-sum test.

**Table 3:** Clinical Outcomes and Postoperative Complications According to Frailty Status.

Characteristic ^2^	Non-Frail Group (N = 133)	Frail Group (N = 308)	Total (N = 441)	*p*-Value ^1^
ICU length of stay (days)	2.0 (1.0, 3.0)	3.0 (1.5, 5.0)	2.0 (1.0, 4.0)	<0.001
Hospital length of stay (days)	17.0 (12.0, 24.0)	24.0 (17.0, 34.0)	22.0 (14.0, 30.0)	<0.001
Any postoperative medical complication	14 (11%)	32 (10%)	46 (10%)	>0.9
Acute kidney injury	0	1	1	>0.9
Significant arrhythmia	13	25	38	>0.9
Respiratory failure or reintubation	1	6	7	>0.9

^1^ Values are presented as median (Q1, Q3), or n (%). ^2^ Wilcoxon rank-sum test, Fisher’s exact test.

**Table 4:** Association Between Postoperative Frailty and Hospital Length of Stay: Minimally Adjusted Model.

Characteristic	IRR	95% CI	*p*-Value
Frail Group	1.38	1.26, 1.51	<0.001
Age	1.01	1.00, 1.01	<0.001
Postoperative Day of Frailty Assessment	1.07	1.05, 1.09	<0.001

IRR: Incidence Rate Ratio, CI: Confidence Interval.

## Data Availability

The original contributions presented in this study are included in the article. Further inquiries can be directed to the corresponding author.
